# Spondylitis in broiler breeder farms in West-Azerbaijan province, Iran: Clinical Report

**Published:** 2016-12-15

**Authors:** Alireza Talebi, Jafar Taifebagherlu, Arian Sharifi, Fatemeh Delkhosh-Kasmaie

**Affiliations:** 1 Department of Poultry Health and Diseases, Faculty of Veterinary Medicine, Urmia University, Urmia, Iran; 2 DVSc Candidate, Department of Poultry Health and Diseases, Faculty of Veterinary Medicine, Urmia University, Urmia, Iran; 3 PhD Candidate, Department of Pathobiology, Faculty of Veterinary Medicine, Urmia University, Urmia, Iran

**Keywords:** Broiler breeder, Iran, Kinky back, Spondylitis, West-Azerbaijan

## Abstract

Spondylitis is a reemerging epidemic spinal infection in male broiler chickens (5 to 7 weeks of age) as well as broiler breeder roosters (15 to 18 weeks of age). Among various causative agents, *Enterococcus* species and in particular *E. cecorum*, a gram-positive bacterium as a gastrointestinal flora of birds, have mostly been isolated. On late September 2015, a number of 10 weeks old roosters with characteristic clinical signs of lameness and hock-sitting posture were autopsied. During thorough general routine post-mortem examinations, abnormalities like nodular masses correlated well with the hock-sitting posture and posterior paresis/paralysis were observed in joint spaces on the caudal thoracic vertebral column (T6-T7) immediately anterior to the kidneys in all affected birds. At histopathological examinations, osteomyelitis with limited pathological lesions including mononuclear inflammatory cells infiltration and edema in spinal cord were seen and the infection was diagnosed as an acute spondylosis.

## Introduction

Lameness together with poor growth was usually seen clinically in breeder farms and male chickens are mostly involved. Among lameness disorders, reemergence of epidemic spinal infection and in particular spondylitis^1^ in male broiler chickens (5 to 7 weeks of age) as well as broiler breeder roosters (3.5 to 18.5 weeks of age) has recently been reported from Europe, North America and South Africa.^1^^-^^7^ Although various causative agents were discriminated, but mostly *Enterococcus* species and in particular *E. cecorum* have been recovered.^4^^-^^8^ Further, different routes of administration of *E. cecorum* experimentally induced spondylitis (ES) referred also as enterococcal vertebral osteoarthritis (EVOA).^5^ As a gram-positive bacterium,* E. cecorum* is also considered as an emerging avian pathogen with significant economic consequences for the poultry industry.^4^^,^^9^^,^^10^ Although, *E. cecorum* could be a gastrointestinal flora^11^ of birds but vertical transmission^12^ appears unlikely and it has been reported that *E. cecorum* isolated from spondylitis may phenotypically and genotypically be different from cecal/ cloacal isolates.^8^

## Case Description

**Fig. 1:**
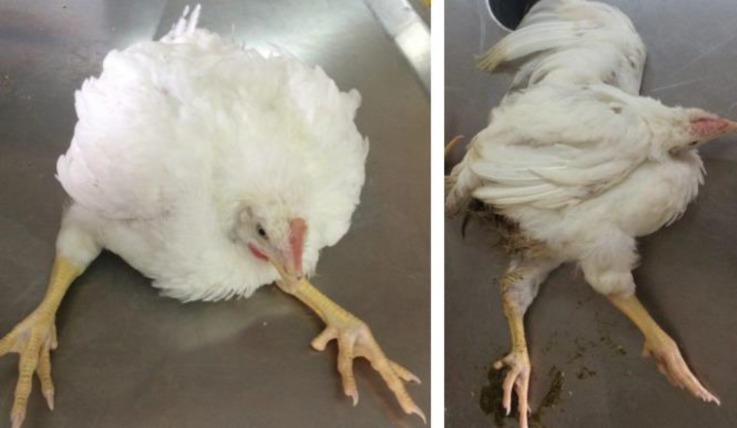
Affected birds sitting on hock and tail with raising feet, often referred as kinky back

On late September 2015, a number of 10 weeks old male birds (Ross-308 broiler breeder, weighing 1570 to 1660 g, estimated morbidity 1.50 to 2.00%) only displayed characteristics of enterococcal ES including posture involving resting on the hocks and paresis to complete posterior paralysis, but swollen joints and tendons did not seen in clinical examinations. Affected birds rested on their hocks and caudal abdomens with legs extended forward and were unable to stand or walk (Fig. 1). This infection may also be referred as EVOA.

**Fig. 2:**
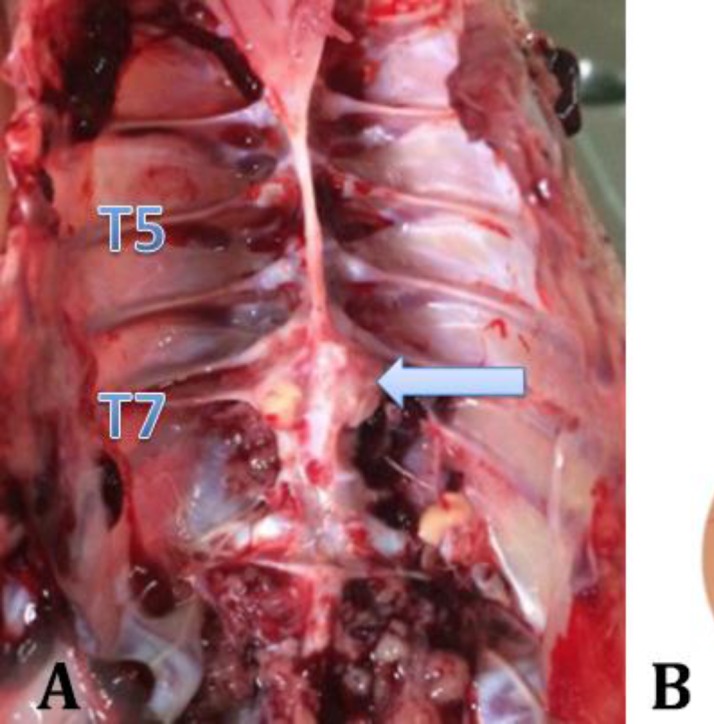
**A) **Sagittal section of vertebral column. Large necrosis area (blue arrow) in T7 vertebra; **B) **Histopathological findings: Mononuclear inflammatory cells (lymphocytes) in pia mater and edema in white matter of spinal cord (H & E; 100× and 400×).

**Necropsy findings. **The birds were stunned and during thorough general routine post-mortem examinations, nodular (firm to hard inflammatory) masses were observed on the caudal thoracic vertebral column (T6-T7) immediately anterior to the kidneys in all affected birds (Fig. 2A). Advanced vertebral osteomyelitis lesions correlated well with the hock-sitting posture and posterior paresis/paralysis were observed. Sagittal cross sections of the vertebral columns revealed vertebral osteomyelitis (a dorsoventral spread of inflammation) induced compression of the overlying spinal cord in the region of T6-T7. Tissue samples from lesions were prepared and sent to Department of Pathobiology, Faculty of Veterinary Medicine, Urmia University, Urmia, Iran.

**Histopathological examination. **In histopathological studies, mononuclear inflammatory cells infiltration consisting of lymphocytes in pia mater of spinal cord and edema in white matter were observed (Fig. 2B). Pathological injuries were limited because of acute spondylosis and the main pathological finding was spinal inflammation.

## Discussion

 Spondylitis in poultry frequently involves the 5^th^ to the 7^th^ thoracic vertebrae (T5-T7), although involvement of 4^th^ thoracic vertebra was also reported.^14^ A possible anatomically explanation for this hinges on the fact that the T2-T5 vertebrae are fused in order to provide rigidity for the structural strength necessary for flight, whilst the T7 vertebra is strongly fused to the lumbosacral vertebrae. Since the sixth thoracic vertebra is the only freely moving articulation in this area, it is subjected to increased mechanical stresses. Weight bearing stress is greater in males than females and in particular roosters in boiler breeder farms have the greatest body weight among chickens. The growth rate in cockerels could be another index affecting body weight in specific period of husbandry in broilers and broiler breeders (4-7 weeks of age and 6-10 weeks of age, respectively) that makes male birds more susceptible for ES infections. *Enterococcus cecorum* as a commensal enteric bacterium is a normal intestinal inhabitant of chickens. Under some conditions including damages to the epithelial cells of intestine or infections and stresses due to growth rate together with physiological intestinal microbial disturbances, *E. cecorum* enters into the blood stream and exerts pathogenic effects in the caudal thoracic vertebrae due to predilection for cartilage and bone.^5^ Spondylitis should be differentiated from spondylolisthesis (kinky back syndrome).^4^^,^^7^ Prevention may include avoiding overstocking, providing adequate feeder space, reducing stress, preventing respiratory diseases and using preventive treatment. Although preventive medications could be more affective,^15^ but resistance of *E. cecorum* to some antibiotics such as lincomycin, tetracycline and erythromycin has been reported.^16^ However, *E. cecorum* could also be sensitive to different antibiotics such as fosfomycin, spectinomycin, enrofloxacin and amoxicillin.^4^ Moreover, antibiogram is recommended for assessment of sensitivity of the isolated pathogenic agents for therapeutic medications. In this case, enrofloxacin 10% at a dose of 10 mg kg^-1^ (500 mL per 1000 L drinking water) for 4 days was recommended.
